# Identification of Conserved and Diverse Metabolic Shift of the Stylar, Intermediate and Peduncular Segments of Cucumber Fruit during Development

**DOI:** 10.3390/ijms19010135

**Published:** 2018-01-03

**Authors:** Chaoyang Hu, Huiyu Zhao, Wen Wang, Mingfei Xu, Jianxin Shi, Xiangbo Nie, Guiling Yang

**Affiliations:** 1 Lab (Hangzhou) for Risk Assessment of Agricultural Products of Ministry of Agriculture, Institute of Quality and Standard for Agricultural Products, Zhejiang Academy of Agricultural Sciences, Hangzhou 310021, China; spiritsun85@163.com (C.H.); zhaohuiyu64@163.com (H.Z.); ww_hi1018@163.com (W.W.); xbrightfly@163.com (M.X.); 2 Key Laboratory of Information Traceability for Agricultural Products of Ministry of Agriculture of China, Institute of Digital Agriculture, Zhejiang Academy of Agricultural Sciences, Hangzhou 310021, China; 3 Joint International Research Laboratory of Metabolic & Developmental Sciences, SJTU-University of Adelaide Joint Centre for Agriculture and Health, School of Life Sciences and Biotechnology, Shanghai Jiao Tong University, Shanghai 200240, China; jianxin.shi@sjtu.edu.cn; 4 Paojiang Jin Bo Family Farm, Shaoxing 312000, China; xbnie@126.com

**Keywords:** metabolite profiling, amino acids, lipids, flavonoids, carbohydrates, PCA, OPLS-DA, correlation analysis

## Abstract

Cucumber (*Cucumis sativus* L.) is one of the most important vegetables and contains a high content of nutritionally beneficial metabolites. However, little is known about the metabolic variations among different parts of cucumber fruit and their kinetics during growth. In this study, the dynamic metabolic profiles in the stylar end, the intermediate segment and the peduncular end of cucumber fruit during the development were investigated by employing a non-targeted metabolomics approach, where 238 metabolites were identified. Statistical analyses revealed that both development time and tissue type influenced metabolic changes, while development time seemed to exert more effects than tissue type on the cucumber fruit metabolome. The levels of the most of the detected metabolites decreased gradually, while those of some amino acids, carbohydrates and flavonoids increased across development. The metabolomes of the stylar end and the intermediate segment were similar, although all three parts of the cucumber fruit were separated from each other in orthogonal partial least squares projection to latent structures-discriminant analysis (OPLS-DA) plots. Metabolites association analysis revealed that *sn*-1 and *sn*-2 lysophospholipids are synthesized via independent pathways in cucumber fruit. In sum, this study demonstrated both conserved and diverse metabolic kinetics of three parts of cucumber fruit, which will facilitate further study of the regulation of cucumber fruit development as well as their potential applications in nutritious quality improvement of cucumber fruit.

## 1. Introduction

Cucumber (*Cucumis sativus* L.), a well-known model plant for the study of sex determination and vascular biology [1,2], is also an important vegetable that is widely cultivated in temperate and tropical regions [3]. China produces the largest proportion of cucumbers, accounting for 75% of the global production in 2013 [4]. Cucumber fruits are normally harvested in fresh and unripe green form approximately 2 weeks after flowering, when they are in the middle-to-late rapid expansion stage [5], since the ripe yellow form is normally bitter and sour. A few cucumber cultivars are parthenocarpic, producing seedless fruit without pollination and making them suitable to grow in greenhouses where bees are excluded.

Although more than 90% of the fresh weight of a cucumber fruit is water, it is important sources of nutrition for human beings and contain cucurbitacins (cucurbitacins A, B, C, D and E), bioactive phenolic compounds [6], and lignans (lariciresinol, pinoresinol, and secoisolariciresinol). These nutritious metabolites are well-known for their diverse pharmacological and biological activities, which include anti-inflammatory, antioxidant, and anti-cancer effects and a reduction of the risk of cardiovascular disease [7,8,9,10,11,12,13,14]. Cucumber fruit is also valuable source of conventional antioxidant nutrients, including β-carotene, vitamin B, vitamin C and manganese [8]. Furthermore, there are still many other beneficial metabolites yet to be explored in cucumber fruits.

Fruit development is a complex and coordinated process coupled with a series of molecular and metabolic changes [15,16]. Cucumber fruit development can be divided into two stages: the early developmental stage (ovary development, cell division and cell expansion) and the maturation stage [5,17]. The developing cucumber fruit represents a major sink for the assimilates in the cucumber plant, and the growing cucumber fruit’s metabolism is important for the partitioning of photosynthate in the cucumber plant. Furthermore, the composition and the content of different metabolites are critical to the quality of the fresh fruit for consumption. In past decades, the metabolisms of developing fruits, including the tomato (*Solanum lycopersicum*) [15,18,19,20,21,22,23], pepino (*Solanum muricatum*) [24], highbush blueberry (*Vaccinium corymbosum*) [25], citrus [26], peach (*Prunus persica*) [16], sweet pepper (*Capsicum annuum*) [27], sweet melon (*Cucumis melo*) [28], grape [29], and strawberry (*Fragaria* × *ananassa*) [30,31], have been widely investigated, and significant changes in metabolite levels were observed. The obvious shift of transcript profile was observed during early cucumber fruit growth [32]. However, we cannot blithely extrapolate these results to other plants such as the cucumber, since great developmental differences were observed not only among different species but even among closely related plants within the same species [33]. Despite the biological and agricultural importance of metabolism in developing cucumber fruit, our current knowledge of the metabolic changes along its development process is not comprehensive, but rather, very limited.

The taste, which is mainly determined by metabolites and volatiles, and the texture of the peduncular end of cucumber fruit are significantly different from those of the stylar end and intermediate segment, but the difference in the nutritious metabolites of these parts is still unknown. The peduncular end connects to the stem, and thus, has priority in obtaining the nutrition translocate in the phloem from source tissue, while the stylar end is furthest away from the stem and receives the nutrition last. It is interesting to investigate whether the metabolic kinetics of these three parts of the cucumber fruit is different along its development. In this study, non-targeted metabolomics was performed to study the metabolism in different segments of a parthenocarpic cucumber (cv. Jinchun 1) fruit during its early development (cell division and expansion stages). The conserved but divergent metabolic shift pattern of the different segments and the metabolic differences among different segments across its development were investigated. The data formed a relatively comprehensive picture of the changes in the chemical composition of both primary and secondary metabolism during the cell division and the expansion stages of cucumber fruit, provided important insights into the underlying metabolic regulation of cucumber development that could facilitate future improvements in cucumber quality.

## 2. Results

### 2.1. Kinetic Metabolic Pattern of a Developing Cucumber Fruit

**Figure 1:**
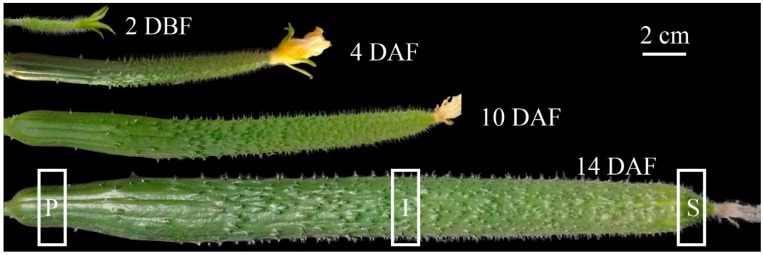
Cucumber fruit at different time points. Samples of the stylar end (S), the intermediate segment (I) and the peduncular end (P) were collected as shown in the figures. DBF, days before flowering; DAF, days after flowering.

The parthenocarpic cucumber, cultivar Jinchun 1 (2*n* = 14), was planted in a greenhouse in Hangzhou. Only one cucumber fruit was kept on each plant and the others were removed to avoid their competition for the nutrition. Four biological replications of the stylar ends, the intermediate segments and the peduncular ends of cucumber fruits at 2 days before flowering (2 DBF) and 4, 10, 14 days after flowering (DAF) were collected (Figure 1). Samples from two cucumber fruits were pooled as one biological replicate. The fine powder of whole fruit was extracted with 80% of methanol solution, which can get a good metabolome coverage [34]. The whole fruit extract was then subjected to a metabolic profiling analysis based on ultra-high-performance liquid chromatography-quadrupole time of flight mass spectrometry (UHPLC-Q-TOF-MS). A total of 238 metabolites with known structures were identified, including 46 amino acids, 33 carbohydrates, 8 cofactors, 19 nucleotides, 47 lipids, 13 benzene derivatives, 66 secondary metabolites (including 43 flavonoids, 21 hydroxycinnamate derivatives, two monoterpenol glycoconjugates) and six other compounds (Table S1).

**Figure 2:**
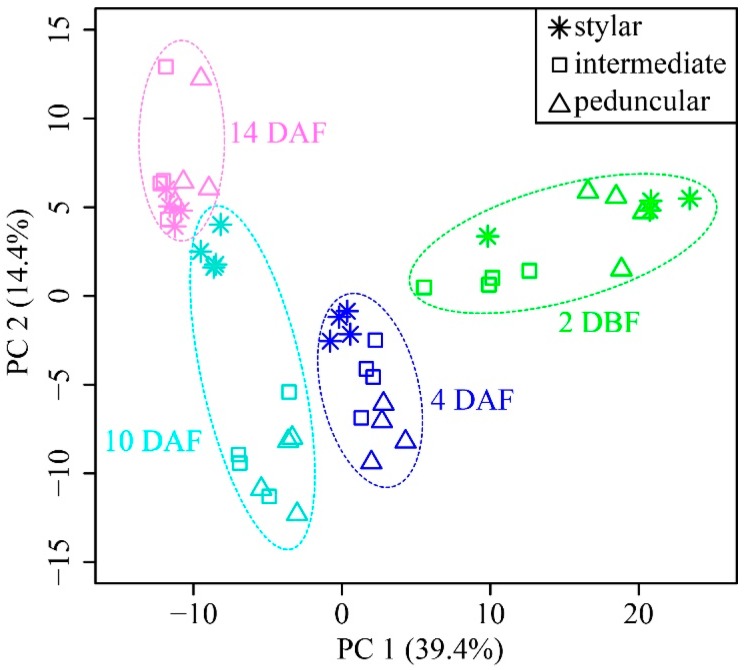
Principal component analysis of the metabolomes of the stylar end, the intermediate segment and the peduncular end of developing cucumber fruit. Green, blue, turquoise and violet colors represent samples at 2 DBF, 4 DAF, 10 DAF and 14 DAF, respectively. Star, square and open triangle denote metabolomes of the stylar end, the intermediate segment and the peduncular end, respectively. The first principal component (PC 1) explains 39.4% of total variance distinguishing cucumber fruit from different developmental time.

A principle component analysis (PCA) on the 238 metabolites was subsequently performed to obtain a global view of the kinetic metabolic patterns of developing cucumber fruits. The first principal component (PC 1), accounting for 39.4% of the total variance, reflected time dependent cucumber development and samples from different segments at the same time point grouped together (Figure 2). This result indicated that developmental time affected cucumber fruit metabolome more than tissue type. Samples of the peduncular ends were separated from the stylar end and the intermediate segments at the same time points in PCA plots (Figure S1), indicating that the metabolomes of the peduncular ends of cucumber fruit were different from those of the other two tissue types at all developmental times.

**Figure 3:**
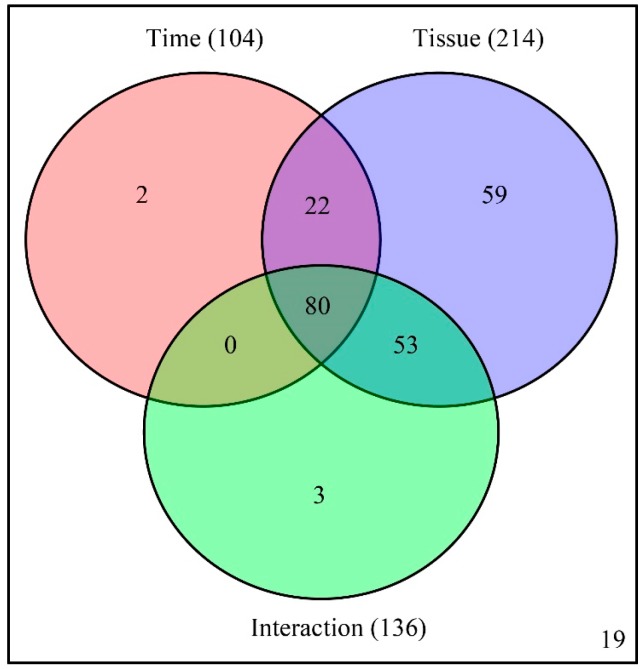
Venn diagram summary of results from two-way ANOVA.

A two-way ANOVA (analysis of variance) was then conducted to determine which factors (developmental time, tissue type, and their interaction) cause the variation of each metabolite. Among the identified 238 metabolites, the abundance of 104, 214, and 136 metabolites were affected by developmental time, tissue type, and their interaction, respectively. Among the metabolites, the abundance of 80 metabolites were simultaneously affected by developmental time, tissue type, and their interactions (Figure 3 and Table S2).

**Figure 4:**
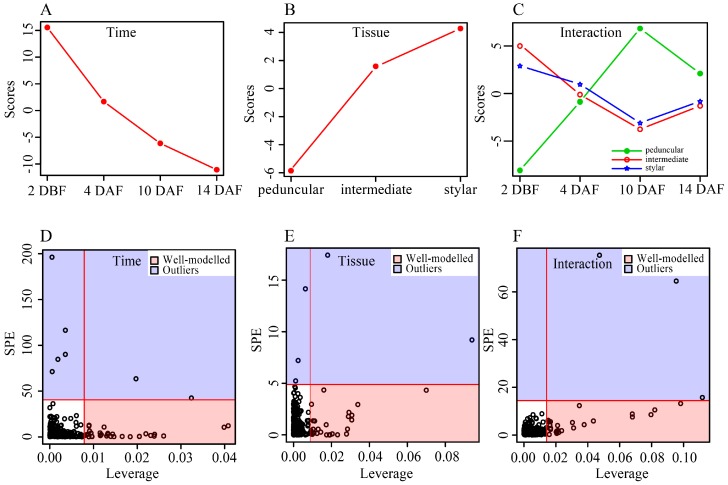
Results of ANOVA simultaneous component analysis (ASCA). (**A**–**C**) Major pattern associated with developmental time, tissue type and their interaction, respectively; (**D**–**F**) ASCA selection of important variables associated with developmental time, tissue type and their interaction by Leverage/SPE analysis, respectively. The list of well-modeled metabolites refer to Table S3.

In addition, an ANOVA-Simultaneous Component Analysis (ASCA) was performed to identify the major patterns with regard to the two given factors and their interaction [35]. Developmental time score plots based on component 1 of the corresponding model showed that the scores gradually decreased along the time (Figure 4A), indicating that the abundance of most metabolites decreased from 2 DBF to 14 DAF. This result was well consistent with the PCA plots shown in Figure 2, in which samples of different tissue types of cucumber fruit shifted in the same direction along its development. The tissue score plot showed that different tissue types differed in their PC1 scores and an increasing gradient was observed from the peduncular end to the stylar end of cucumber fruit (Figure 4B). Component 1 of the interaction effect clearly showed the same trends over all of the time points between the intermediate segment and the stylar end while the peduncular end showed the opposite trend (Figure 4C). The distances among the three tissue types of cucumber fruit at 4 and 14 DAF were relatively small, while the peduncular end was away from the intermediate segment and the stylar end at the other two time points in the interaction score plot (Figure 4C), suggesting that the differences between the interaction effects among these tissues were relatively smaller at 4 and 14 DAF than those at 2 DBF and 10 DAF. This result was also consistent with the PCA score plots shown in Figure 2, in which the stylar ends, the intermediate segments and the peduncular ends grouped tighter in 4 and 14 DAF groups than those at the other two time points.

Leverage/squared prediction error (SPE) plots were made to correlate the metabolic features with the experimental factors [36]. Leverage evaluates the importance of the metabolite to the model, and SPE tests the fitness of the model for particular metabolites. Metabolites with high leverage and low SPE that contributed significantly to the model were picked out as well-modeled metabolites. Thirty well-modeled metabolites, including 25 flavonoids, stood out based on the major pattern of time (Figure 4D and Table S3), and the levels of these metabolites gradually decreased in all three tissues of the cucumber fruit. A total of 29 metabolites, including 12 flavonoids and five amino acids, were well-modeled by tissue type (Figure 4E and Table S3), the abundance of which differed among tissues. For example, the levels of astilbin, caffeic acid-*O*-hexoside II, chalcone 2′-*O*-glucoside, isovitexin and kaempferol-3-*O*-rhamnoside increased from 2 DBF, peaked at 10 DAF, then sharply declined at 14 DAF in both the stylar end and the intermediate segment, but they tended to remain stable in the peduncular end (Figure S2). A total of 25 metabolites, including 18 flavonoids and five amino acids, stood out based on the major pattern of interactive effect (Figure 4F and Table S3), and the levels of these metabolites changed differently among tissue types along development of the cucumber fruit.

### 2.2. Metabolic Changes along Cucumber Fruit Development

To investigate the change patterns of each metabolite along cucumber fruit development, the levels of the 238 identified metabolites at 4 DAF, 10 DAF and 14 DAF were compared with those at 2 DBF of the same tissue to eliminate the tissue-dependent variation. 

**Figure 5:**
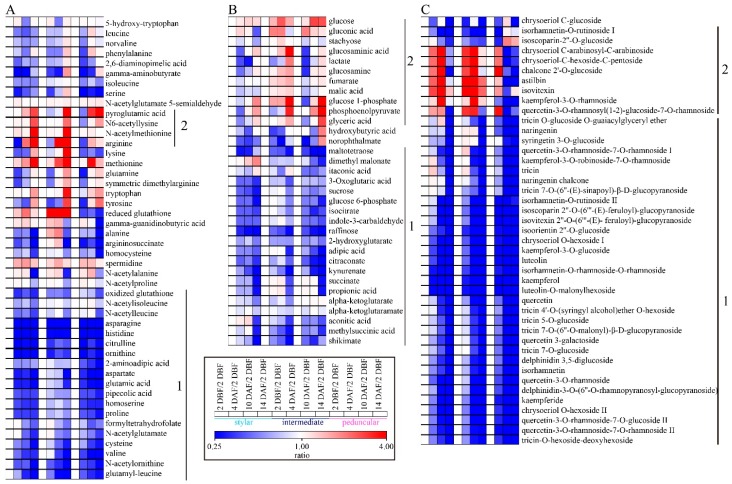
Heat map of metabolite changes in the stylar end, the intermediate segment and the peduncular end during cucumber fruit development. (**A**) amino acids; (**B**) carbohydrates; (**C**) flavonoids. Ratios of fold changes were given by shades of red or blue colors according to the scale bar. Ratios were calculated as follows: the mean values from four biological replicates at each time point were divided by those at 2 DBF of the same tissue type to eliminate the tissue-dependent variation.

Various kinetic metabolic change patterns were observed in the amino acids (Figure 5A). The abundances of 17 amino acids (Figure 5A, label 1), such as aspartate, asparagine, glutamic acid, *N*-acetylglutamte, citrulline, ornithine, *N*-acetylornithine and oxidized glutathione, significantly decreased, while those of methionine, tryptophan and spermidine increased in all three different parts of the cucumber fruit along its development. The levels of four amino acids, pyroglutamic acid, arginine, *N*-acetyllysine and *N*-acetylmethionine significantly increased, mainly at 14 DAF, in all three different parts of cucumber fruit, especially in the stylar end and the intermediate segment (Figure 5A, label 2). Notably, the dynamic change patterns of some amino acids were tissue-specific. For example, the level of reduced glutathione significantly decreased in the peduncular end while it slightly increased in the stylar end and strongly increased in the intermediate segment, respectively.

The levels of 20 carbohydrates, such as sucrose, raffinose, glucose 6-phosphate and maltotetraose, decreased gradually in all three different parts of the cucumber except that of dimethyl malonate in the stylar end, which increased gradually along the cucumber fruits’ development (Figure 5B, label 1). The abundances of 11 carbohydrates, such as glucose, glucose 1-phosphate and phosphoenolpyruvate (PEP), tended to increase slightly (Figure 5B, label 2). The variations in the levels of TCA cycle intermediates, such as fumarate, malic acid, succinate, and alpha-ketoglutarate, were relative small along cucumber fruit development, except that of succinate, which decreased significantly at 14 DAF.

The levels of seven lipids (including five fatty acids, myristic acid, linoleic acid, linolenic acid, pinolenic acid and 2-hydroxypalmitate) decreased gradually in all three different parts of the cucumber (Figure S3A, label 1). The levels of the majority (16 out of 20) of the lysoglycerophosphatidylcholines (LysoPCs) and lysoglycerophosphatidylethanolamines (LysoPEs) started to decrease from 4 DAF in the stylar and the peduncular ends while they decreased from 10 DAF in the intermediate segment (Figure S3A, label 2). The levels of six lipids (including four oxolipids) decreased at 4 DAF and 10 DAF in all three different parts of the cucumber, while they were not significantly changed or even increased slightly at 14 DAF in the stylar end and the intermediate segments, only (Figure S3A, label 3). The change patterns of three monoacylglycerides (i.e., 1-palmitoylglycerol, 2-palmitoylglycerol and 2-linoleoylglycerol, were tissue specific) increased gradually in the intermediate segment, while they decreased in the peduncular end during cucumber development (Figure S3A, label 4).

The general kinetic change trend of the levels of cofactors, nucleotides, hydroxycinnamate derivatives and benzene derivatives was similar to that of lipids, which decreased gradually along cucumber fruit development (Figure S3B–S3E). However, the levels of some metabolites of these classes increased in some tissues at some time points. For example, the levels of ascorbate and dehydroascorbate increased slightly at 4 DAF and 10 DAF in the stylar end (Figure S3B). The levels of adenine, guanosine 3′,5′-cyclic phosphate, guanosine 5′-phosphate and uridine monophosphate increased significantly in the intermediate segment (Figure S3C). The abundance of indole increased highly in all segments of cucumber fruits at 4, 10 and 14 DAF compared to that at 2 DBF, while that of 3-indoleacetic acid increased only in the stylar end (Figure S3D). The levels of caffeic acid-*O*-hexoside II increased at 4 and 10 DAF in both the stylar end and the intermediate segment of cucumber fruits (Figure S3E).

The dynamic change patterns of two isomers of monoterpenol glycoconjugates were different though both levels tended to increase along cucumber fruit development (Figure S3F). The level of terpenyl-pentosyl-glucoside I peaked at 4 DAF and then gradually decreased along cucumber development; however, it was still significantly higher at 10 DAF and 14 DAF than at 2 DBF in all three different parts except at 14 DAF in the peduncular end. The levels of terpenyl-pentosyl-glucoside II increased gradually and peaked at 14 DAF in the stylar end, while the levels fluctuated within a relatively narrow range in the intermediate segment and the peduncular end. Accumulation of monoterpenol glycoconjugates in cucumber fruit along development is associated with age-related resistance to pathogenic microorganism, such as *Phytophthora capsici* [37].

The levels of 76.7% (33 out of 43) of flavonoids, such as tricin, luteolin, kaempferol, quercetin, isorhamnetin and their glycosides, decreased gradually along development in all segments of the cucumber fruit (Figure 5C, label 1). Nine flavonoids showed the same change patterns in both the stylar end and the intermediate segment but different from those in the peduncular end (Figure 5C, label 2). For example, the levels of isorhamnetin-*O*-rutinoside I and isoscoparin-2″-*O*-glucoside gradually decreased in both the stylar end and the intermediate segments but were not significantly changed or even slightly increased in the peduncular end at 10 and 14 DAF compared to those at 2 DBF. The levels of chrysoeriol *C*-arabinosyl-*C*-arabinoside, chrysoeriol-*C*-hexoside-*C*-pentoside, chalcone 2′-*O*-glucoside, astilbin, isovitexin, kaempferol-3-*O*-rhamnoside and quercetin-3-*O*-rhamnosyl(1-2)-glucoside-7-*O*-rhamno-side increased from 2 DBF and peaked at 10 DAF in both the stylar end and the intermediate segment, while the level increased only at 4 DAF and then significantly decreased at 10 and 14 DAF in the peduncular end.

### 2.3. Metabolic Difference among Tissues at Each Time Point

**Figure 6:**
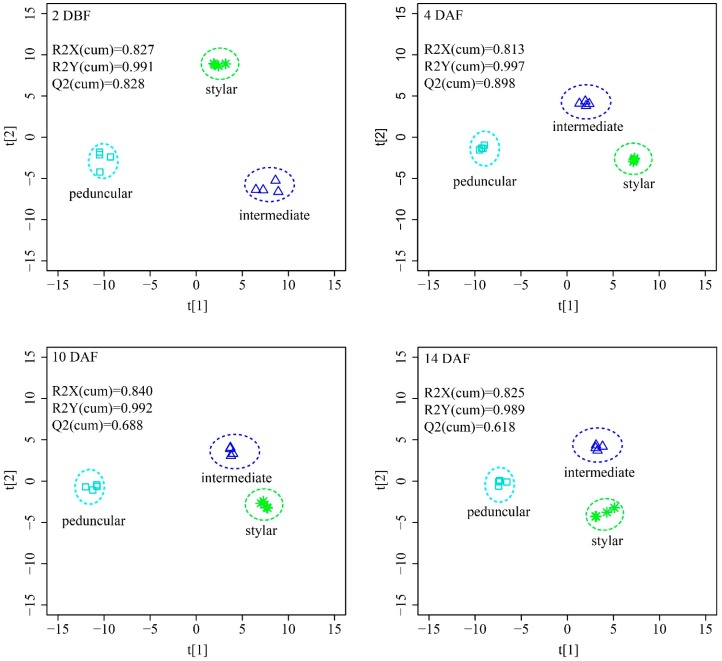
OPLS-DA score plots discriminating stylar end, intermediate segment and peduncular end of cucumber fruit from each other at all the four time points.

To obtain maximal covariance between the metabolite levels and the tissues of the cucumber fruit at each time point, an orthogonal partial least squares projection to latent structures-discriminant analysis (OPLS-DA) was applied. The OPLS-DA model established with two predictive components and three orthogonal components generated, the explained variation values: R2X(cum) > 0.82, and R2Y(cum) > 0.98 and the predictive capability: Q2(cum) > 0.62 at all four time points (Figure 6). These high value parameters indicated the excellence in modeling and prediction with good discrimination among the stylar end, the intermediate segment and the peduncular end of cucumber fruit since OPLS-DA models with parameters higher than 0.5 are considered to be satisfactory in explanatory and predictive capabilities [38]. The score plots of OPLS-DA showed a distinct separation among the three tissues at all four time points (Figure 6). The stylar ends and the intermediate segments located at the same side of the score plots, while the peduncular ends on the other side indicated that the metabolic profiles between the stylar ends and the intermediate segments were more similar than those of the peduncular ends.

Based on a variable importance in projection (VIP) threshold (VIP > 1.5) from the seven-fold cross-validated OPLS-DA models, a number of metabolites responsible for the differentiation of metabolic profiles of the stylar end, the intermediate segment and the peduncular end of cucumber at each time point were obtained. In parallel, the candidate metabolites from OPLS-DA model, whose mean values significantly (false discovery rate < 0.05) differed among the tissues of cucumber fruit tested by one-way analysis of variance (ANOVA) with post hoc Tukey’s honestly significant difference (HSD), were further selected as biomarkers (Table 1).

At 2 DBF, a total of 14 metabolites, including four amino acids, one benzene derivative, one cofactor, six flavonoids, one hydroxycinnamate derivatives and one nucleotide were selected as biomarkers (Table 1). The levels of most of these metabolites were significantly different between the peduncular ends and the other two tissues but were not significantly different between the stylar end and the intermediate segment. The relative abundances of guanosine 5′-diphosphate, tricin *O*-glucoside *O*-guaiacylglyceryl ether, valine, quercetin-3-*O*-rhamnoside-7-*O*-rhamnoside II and 3-indoleacetic acid were the highest in the peduncular end compared with those in the stylar end and the intermediate segments. In contrast, the levels of isoscoparin 2″-*O*-(6″-(E)-feruloyl)-glucopyranoside, histidine, isorhamnetin-*O*-rutinoside II, isovitexin 2″-*O*-(6″-(E)-feruloyl)-glucopyranoside and isorhamnetin-*O*-rutinoside I were the lowest in the peduncular end. The levels of four metabolites, i.e., ascorbate, aspartate, serine and coumaric acid hexoside III, were significantly different between the stylar end and the intermediate segment.

At 4 DAF, a total of 14 metabolites, including one carbohydrate, nine flavonoids, two hydroxycinnamate derivatives and two monoterpenol glycoconjugates, were important in distinguishing the three different tissues of the cucumber fruit from each other (Table 1). The levels of terpenyl-pentosyl-glucoside I and feruloylquinic acid II in the stylar end were the highest compared with those in the intermediate segment and the peduncular end. The highest level of tricin *O*-glucoside *O*-guaiacylglyceryl ether, while the lowest levels of isoscoparin 2″-*O*-(6″-(E)-feruloyl)-glucopyranoside, isorhamnetin-*O*-rutinoside II, isovitexin 2″-*O*-(6″-(E)-feruloyl)-glucopyranoside, luteolin, kaempferol-3-*O*-glucoside and luteolin-*O*-malonylhexoside, were found in the peduncular ends. The abundances of terpenyl-pentosyl-glucoside II and norophthalmate were relatively higher in the intermediate segment, while those of tricin 7-*O*-(6″-(E)-sinapoyl)-β-d-glucopyranoside and kaempferol-3-*O*-robinoside-7-*O*-rhamnoside were the lowest in the intermediate segment of the cucumber fruit.

At 10 DAF, a total of 15 metabolites, including one benzene derivative, two carbohydrates, one cofactor, six flavonoids, one hydroxycinnamate derivative, three lipids and one monoterpenol glycoconjugates, had important roles in separating the three different parts of the cucumber fruit from each other. The levels of the top five metabolites with the highest VIP values (astilbin, caffeic acid-*O*-hexoside II, isovitexin, chalcone 2′-*O*-glucoside and terpenyl-pentosyl-glucoside I), two lipids (linolenoyl ethanolamide and phytosphingosine) and the other three flavonoids (chrysoeriol-*C*-hexoside-*C*-pentoside, chrysoeriol *C*-arabinosyl-*C*-arabinoside and kaempferol-3-*O*-rhamnoside) were all higher in the stylar end and the intermediate segment than those in the peduncular ends. The levels of sphinganine and 3-indoleacetic acid were significantly different in all three tissues, with the highest level of 3-indoleacetic acid and the lowest level of sphinganine in the peduncular ends. The levels of two carbohydrates, i.e., phosphoenolpyruvate and methylsuccinic acid, were significantly higher in the stylar end than those in the intermediate segment and the peduncular end.

At 14 DAF, a total of eight metabolites, including six amino acids, one flavonoid and one monoterpenol glycoconjugate were present (Table 1). The levels of all the eight metabolites were the lowest in the peduncular end. Similar high levels of lysine, serine and kaempferol-3-O-rhamnoside were found in the stylar end and the intermediate segment. The levels of valine, terpenyl-pentosyl-glucoside II and gamma-guanidinobutyric acid in the stylar end were significantly higher than those in the intermediate segment and the peduncular end. The highest abundance of methionine was observed in the intermediate segment.

### 2.4. Metabolite-Metabolite Correlations in Developing Cucumber Fruit

As described above, the majority of the metabolites were down-regulated, though some of them were up-regulated, during the early development of cucumber fruit. Thus, it is interesting to investigate which metabolites were co-regulated to better understand the metabolic regulation process in cucumber fruit and to look for a possible conserved metabolic pathway for metabolic engineering to increase the nutritional metabolite levels in the future. To this end, a pair-wise correlation analysis was performed by employing the Pearson’s product-moment correlation with the 238 identified metabolites. The correlation coefficients and *p*-values are affected by the sample number. Normally, a larger sample number can provide more reliable correlations, though with lower coefficients. Thus, the metabolite levels of 48 individual samples (i.e., each metabolite has 48 values), but not the mean metabolite levels of four biological replications, were used to obtain more reliable correlations.

**Figure 7:**
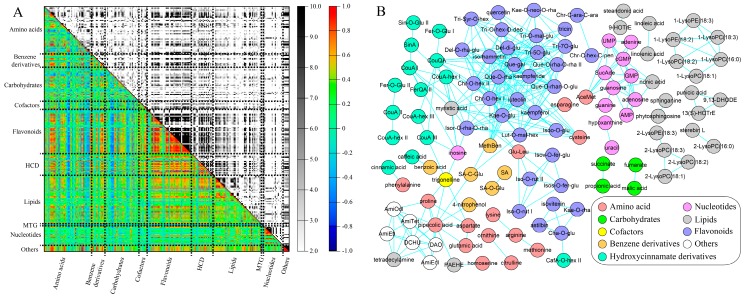
Metabolite-metabolite correlation in developing cucumber fruit. (**A**) Heatmap of all results of metabolite-metabolite correlation and significance. In the colored area, rectangles represent Pearson correlation coefficient (*r*) values of metabolite pairs (see correlation color key). In the black and white area, rectangles represent the respective *p*-values (see significance color key). Details about the associations are listed in Table S4; (**B**) Highly associated (*r*-value > 0.90) metabolite-metabolite correlations. Full names of the abbreviation of metabolites refer to Table S1. Each color denotes a compound class as shown in the bottom right legend.

At the threshold of an absolute correlation coefficient greater than 0.70 (|*r*-value| ≥ 0.70), there were 2989 pairs of positive correlations and 29 pairs of negative correlations (Table S4). These positive correlations were found in all metabolite classes while most of them were associated with flavonoids, hydroxycinnamate derivatives, benzene derivatives or lipids (Figure 7A). This is consistent with the abovementioned metabolic change patterns in cucumber fruits in which the levels of these metabolites decreased in all three different segments of the cucumber fruit during its early development. Six, five and four negative correlations were found to be connected with quinate, pyroglutamic acid and glutamine, respectively. Levels of these three metabolites increased gradually along cucumber development. Almost all of the highly correlated metabolite pairs (*r*-value > 0.90) belonged to the same compound class, and the majority of them were flavonoids (Figure 7B and Table S4), indicating that metabolites involved in the same metabolic pathway were highly co-regulated.

The highly correlated metabolite pairs were observed between metabolites with at least three biological relations (Table 2). The first relation was metabolites with the substrate–product connection in a metabolic reaction, such as phenylalanine and cinnamic acid, fumarate and malate, linoleic acid and linolenic acid, coumaric acid and coumaric acid hexoside, luteolin and luteolin-*O*-malonylhexoside, etc. The second relation concerned isomers, such as chrysoeriol *O*-hexoside I and chrysoeriol *O*-hexoside II, kaempferol and luteolin, tricin 5-*O*-glucoside and tricin 7-*O*-glucoside, etc. The third relation represented metabolites with a similar structure or belonging to the same sub-class, which was clearly observed in lipids. For example, linoleic acid and linolenic acid (unsaturated fatty acids), 9,13-DHODE and 13(s)-HOTrE (oxolipids), LysoPEs and LysoPCs (glycerophospholipids) were highly correlated (Figure 7B). It is interesting that LysoPEs and LysoPCs with the fatty acyl chain at the same position of glycerol (*sn*-1 or *sn*-2, *sn* means stereospecific numbering), were highly correlated. For example, 1-LysoPE(18:3), 1-LysoPE(18:2), 1-LysoPC(18:3), 1-LysoPC(18:2), 1-LysoPC(18:1) and 1-LysoPC(16:0) were highly correlated, while 2-LysoPC(18:3) was highly associated with 2-LysoPE(18:3), 2-LysoPC(18:2) and 2-LysoPC(18:1).

## 3. Discussion

Fruit development is a highly complex and coordinated process that has gained additional research attention related to its metabolic aspects in recent years [39]. Investigation into the metabolic profile during fruit development is necessary to gain a better understanding of the complex process. Metabolites that accumulate in fruit during development largely contribute to the fruit quality traits, including fruit taste and texture [21]. Developmental profiles of carbohydrate metabolism in cucumber have previously been reported in three studies [40,41,42], but only a limited number of sugars (sucrose, raffinose, stachyose, verbascose, glucose, fructose and galactose) were covered by these studies. In the present study, a broader analysis was conducted by means of an UHPLC-Q-TOF-MS approach, which covered a more comprehensive range of metabolites investigated in the three different segments (the stylar end, the intermediate segment and the peduncular end) of cucumber fruit. 

The abundance of most of the metabolites decreased along cucumber fruit development during its early development (i.e., cell division and expansion stages), and some similar metabolic shift patterns were observed in other fruits. For example, a major decrease in the levels of carbohydrates and amino acids in strawberry and tomato and that of flavonoids in apple and cranberry, were observed during their early developmental stages [19,30,31,43,44]. The finding indicated that the metabolic regulation of the fundamental processes of fruit in early development may be conserved among species [19]. Some opposite metabolic change patterns have also been observed in other fruits, compared with those in cucumber fruit in this study. For example, the level of sucrose highly increased in peaches [16], and the levels of fatty acids in strawberry fruit were relative stable [31] during the early developmental stage while they significantly decreased in cucumber fruit. This suggested that the regulation of some metabolic pathways in fruits during their early developmental stage was species specific.

The metabolic profiles of the stylar end, the intermediate segment and the peduncular end were significantly different throughout cucumber fruit development, suggesting that each part of cucumber fruit has some unique structure and physiology. This result is logical because the metabolome is tissue-, cell- and compartment-specific [45]. However, contrary to our expectations, most of the differential metabolites among these three parts of cucumber fruit had the lowest levels in the peduncular end (Table 1), though it had priority to absorb nutrition from phloem, suggesting that the intermediate segment and the stylar end were the primary sink tissues during cucumber fruit development. Despite the difference in metabolite levels, the dynamic change patterns of metabolite profile of the stylar end, the intermediate segment and the peduncular end were similar (Figure 2), indicating that the development of different parts of cucumber fruit was highly coordinated.

The association of metabolites through development is determined by the degree of similarity in their accumulation patterns. Because the levels of many metabolites decreased simultaneously along cucumber fruit development, the associated metabolites were not necessarily connected in metabolic pathways. When we focused on those correlations that showed coefficients above 0.90, most of the correlations were observed between metabolites of the same compound class (Figure 7b), which is reasonable because many metabolites in the same pathway were highly co-regulated. Similar phenomena were observed in the developing receptacle of strawberries [31] and tomato fruits [19]. However, the highly correlated metabolite pairs were different among species because few of the metabolites identified in this study were the same as those detected in strawberries and tomatoes. Furthermore, too many correlations were produced in each study, so it is very difficult to compare the correlation results between the different studies in detail. Up to date, the interesting correlation relationships in metabolites of lysophospholipids observed in this study were not revealed before. 

Lysophospholipids (including LysoPEs and LysoPCs) are vital to all organisms and have two kinds of isobars with the aliphatic chains that are connected to *sn*-1 or *sn*-2 of glycerol [46]. However, the biosynthesis pathway is still not very clear, especially in plants. In this study, *sn*-1 LysoPEs and LysoPCs were highly correlated with each other and *sn*-2 LysoPEs and LysoPCs were tightly correlated. However, *sn*-1 lysophospholipids were not highly correlated with *sn*-2 lysophospholipids. We deduce that *sn*-1 and *sn*-2 lysophospholipids must be synthesized from two independent metabolic pathways in cucumber fruit. There are two findings supporting this theory: (1) LysoPC acyltransferase 3 (LPCAT3), LPCAT4 and LysoPE acyltransferase are responsible for the synthesis of *sn*-2 lysophospholipids but not *sn*-1 lysophospholipids in mammalians [47]; and (2) in the triacylglycerol biosynthesis pathway, glycerol-3-phosphate acyltransferase (GPAT) can catalyze glycerol-3-phosphate and FA-CoA into CoA and *sn*-1 lysophosphatidic acids [48], which may be the substrates of the other *sn*-1 lysophospholipids, including LysoPEs and LysoPCs. 

This study provided significant information for comprehending what the metabolite composition of the fresh cucumber fruit is and revealed novel insights into the underlying developmental shifts in primary and secondary metabolism in cucumber fruit. In the future, metabolite profiling will be integrated with transcriptome, proteome, enzyme activity analysis and metabolic flux analysis, thus facilitating cognition of the important and complex process of cucumber fruit development. 

## 4. Materials and Methods

### 4.1. Materials

The seeds of *Cucumis sativus* L. cv. Jinchun 1, one parthenocarpic cultivar, were soaked in warm water (55 °C) for 15 min to promote germination and was put into seedling-raising disk for 25 days. The seedlings were transplanted into a greenhouse in Hangzhou (31.03° N, 121.45° E), Zhejiang province, China, in September 2016. One week before transplanting, the soil was fertilized with 700 kg/ha of compound fertilizer (YaraMila, Oslo, Norway) and 4000 kg/ha of organic fertilizer (Baicheng, China) and one day before transplanting, the soil was treated with fungicide DuPont^TM^ Curzate^R^ (Shanghai DuPont Agro-Chemical Ltd., Co., Shanghai, China), the application concentration was 600 dilution times. Seven days after transplanting, the plants were sprayed with 80% mancozeb and 10% thiophanate-methyl, the application concerntration was 600 dilution times and 1000 dilution times respectively to prevent downy mildew. Forty days after transplanting, the plants were treated with 40 kg/ha of compound fertilizer and 70 kg/ha of urea.

Only one cucumber fruit was kept on each plant and any other fruits were removed to avoid their competition for the nutrition. Samples from two cucumber fruits were pooled as one biological replication. Four biological replications of the stylar ends, the intermediate segments and the peduncular ends of whole cucumber fruit, including mesocarp, pericarp and locular tissue, at 2 DBF, 4 DAF, 10 DAF and 14 DAF were collected, immediately frozen with liquid nitrogen, lyophilized for 48 h and stored at −80 °C until metabolomics analysis.

### 4.2. Metabolite Profiling

Samples were grounded into a fine powder. Each 20 mg of fine powder was used for metabolite extraction prior to UHPLC-Q-TOF-MS analysis. The metabolite extraction procedure was carried out after adding 500 µL of a mixture of methanol and water (4:1) into the fine powder. After vortexing for 3 min, the mixture was kept at room temperature for 10 min and then centrifuged at 13,000× *g* for 10 min. The supernatant was filtered through a syringe filter (0.22 µm) and placed into the sampling vial, pending UHPLC-Q-TOF-MS analysis in both positive and negative mode.

The liquid chromatography analysis was performed on the Agilent 1290 Infinity II LCTM system. A 1.0-µL aliquot of the filtrate was injected into an Agilent Eclipse-plus C18 column (150 × 3.0 mm i.d., 1.8 μm), and the column temperature was set at 30 °C. The mobile phase consisted of A (0.1% formic acid in water) and B (100% acetonitrile) for both positive and negative ion mode. The column was eluted with gradient conditions of the mobile phase as follows: 0 min, 98.0% A; 1.0 min, 98% A; 5.0 min, 60% A; 12.0 min, 30% A; 15.0 min, 5% A; 20.0 min, 5% A. The flow rate was 0.40 mL/min. All the samples were kept at 4 °C during the analysis.

The mass spectrometric data was collected using an Agilent 6545 Q-TOF MS (Agilent Technologies Inc., Palo Alto, CA, USA) equipped with an electrospray ionization (ESI) source. The ESI source was operated in positive and negative ionization modes with a capillary voltage of 3.5 kV for both modes, nozzle voltage at 500 V (+) and 1500 V (−), fragmentor voltage of 110 V, nebulizer at 45 psi, sheath gas and dry gas set at the flow rate of 8 L/min and 8 L/min, respectively, and CID voltage applied with 10, 30 and 50 eV. Centroid data was recorded from 50 to 1100 *m/z* with a scan rate at 2 spectra/s.

The metabolites were annotated by searching the Personal Compound Database and Library (PCD/PCDL), which was established in our previous study [49], and by comparing the MS and MS/MS of the compounds detected in cucumber fruit with those in the Metlin database [50] and the Massbank database [51]. Data acquisition, metabolite annotation and peak area extraction were performed with the Agilent softwares (Agilent Technologies Inc., Palo Alto, CA, USA), of MassHunter Acquisition 7.0, MassHunter Qualitative 7.0 and Mass Profinder 8.0, respectively. 

### 4.3. Data Analysis

The sample weight and non-normalized peak areas are available in Table S5. For data normalization, peak areas were divided by the sample weight and the median value of each metabolite. The missing values of a giving metabolite were imputed with the detected minimum value of the same metabolite for statistical analysis, assuming that they were below the limits of instrument detection sensitivity. The final statistics matrix, with normalized data for the following statistical analysis, is available in Table S1.

A PCA and an OPLS-DA was performed with the SIMCA-P 13.0 software (Umea, Sweden) package using Pareto scaling. For OPLS-DA, the default 7-round cross-validation was applied with 1/7th of the samples being excluded from the mathematical model in each round, to guard against over-fitting. A one-way ANOVA, a post hoc analysis, a two-way ANOVA and an ASCA were carried out in MetaboAnalyst 3.0 website (http://www.metaboanalyst.ca/) according to its protocol [35,52] and “Pareto scaling” was selected as data scaling method for all of these analyses. A within-subjects two-way ANOVA was used, and the significance threshold was defined as the corrected *p*-value < 0.05. The False Discovery Rate was chosen for multiple testing correction. For ASCA, the leverage threshold and alpha threshold were set to be 0.8 and 0.05, respectively. A metabolite-metabolite correlation analysis was performed by employing the Pearson’s product-moment correlation with individual profile values in the R package. The heatmaps of the metabolite ratios and metabolite-metabolite correlations were visualized with MultiExperiment Viewer (MeV) version 3.5.1 (J.Craig Venter Institute, Rockville, MD, USA). The figures were edited with Adobe Illustrator CS5 software (Adobe, San Jose, CA, USA) for better resolution.

## Figures and Tables

**Table 1:** Summary of metabolites that significantly contribute to the discrimination among the stylar end, intermediate segment and peduncular end of cucumber fruit at each time point.

Time Points	Metabolite Name	VIP ^a^	FDR ^b^	Post-Hoc ^c^	Metabolite Class ^d^	Relative Content ^e^		
						Stylar	Intermediate	Peduncular
2 DBF	guanosine 5′-diphosphate	3.52	0.0252	P-I	N	10.57 ± 5.11	4.40 ± 1.46	22.8 ± 9.65
	tricin *O*-glucoside *O*-guaiacylglyceryl ether	3.46	0.0001	P-I; P-S	F	1.27 ± 0.35	1.83 ± 0.50	13.99 ± 2.33
	isoscoparin 2″-*O*-(6″-(E)-feruloyl)-glucopyranoside	2.31	0.0093	I-P; S-P	F	8.96 ± 2.18	10.54 ± 2.18	3.45 ± 1.20
	histidine	2.11	0.0219	S-P	A	8.13 ± 2.44	5.95 ± 1.98	2.44 ± 0.87
	isorhamnetin-*O*-rutinoside II	2.04	0.0194	I-P; S-P	F	6.03 ± 2.77	7.15 ± 1.82	1.39 ± 0.35
	isovitexin 2″-*O*-(6″-(E)-feruloyl)-glucopyranoside	1.99	0.0049	S-P; I-P	F	6.15 ± 1.23	5.77 ± 1.35	1.57 ± 0.44
	valine	1.66	0.0093	P-I; P-S	A	3.27 ± 0.90	2.07 ± 0.66	5.84 ± 1.29
	ascorbate	1.65	0.0076	I-P; I-S	Co.	0.98 ± 0.75	3.82 ± 0.99	1.61 ± 0.24
	quercetin-3-*O*-rhamnoside-7-*O*-rhamnoside II	1.65	0.0378	P-I; P-S	F	4.79 ± 0.48	4.46 ± 1.06	8.26 ± 2.52
	aspartate	1.64	0.0076	P-I; S-I	A	3.88 ± 1.25	1.39 ± 0.18	4.56 ± 0.63
	serine	1.61	0.0069	S-I; S-P	A	3.75 ± 0.74	1.29 ± 0.55	1.89 ± 0.47
	isorhamnetin-*O*-rutinoside I	1.60	0.0083	I-P; S-P	F	3.85 ± 1.02	3.92 ± 1.09	0.89 ± 0.16
	3-indoleacetic acid	1.55	0.0235	P-S	B	0.30 ± 0.00	1.40 ± 0.81	3.42 ± 1.64
	coumaric acid hexoside III	1.54	0.0076	P-I; S-I	H	4.34 ± 0.71	1.88 ± 0.79	3.64 ± 0.16
4 DAF	terpenyl-pentosyl-glucoside I	6.49	0.0029	S-I; S-P	M	30.8 ± 6.52	12.2 ± 7.55	3.55 ± 1.92
	tricin *O*-glucoside *O*-guaiacylglyceryl ether	2.95	1.70E-06	P-I; P-S	F	1.09 ± 0.29	1.66 ± 0.34	7.47 ± 0.61
	isoscoparin 2″-*O*-(6″-(E)-feruloyl)-glucopyranoside	2.65	0.0037	I-P; S-P	F	7.51 ± 2.27	6.15 ± 1.62	0.66 ± 0.29
	isorhamnetin-*O*-rutinoside II	2.34	0.0179	I-P; S-P	F	5.47 ± 2.26	4.86 ± 2.01	0.38 ± 0.20
	caffeic acid-*O*-hexoside II	2.25	0.0308	S-P	H	5.23 ± 2.01	2.67 ± 1.11	1.41 ± 0.54
	tricin 7-*O*-(6″-(E)-sinapoyl)-β-d-glucopyranoside	2.10	0.0163	P-I; S-I	F	1.80 ± 0.30	0.99 ± 0.38	2.28 ± 0.49
	isovitexin 2″-*O*-(6″-(E)-feruloyl)-glucopyranoside	2.10	0.0043	I-P; S-P	F	4.97 ± 1.53	3.80 ± 1.17	0.35 ± 0.17
	terpenyl-pentosyl-glucoside II	2.07	0.0173	I-S	M	0.28 ± 0.35	1.30 ± 0.28	0.73 ± 0.31
	kaempferol-3-*O*-neohesperidoside-7-*O*-rhamnoside	1.93	0.0287	P-I; S-I	F	3.42 ± 0.36	2.58 ± 0.12	3.20 ± 0.35
	luteolin	1.81	0.0043	I-P; S-P	F	3.32 ± 0.15	3.78 ± 0.36	2.39 ± 0.41
	kaempferol-3-*O*-glucoside	1.76	0.0022	I-P; S-P	F	3.02 ± 0.05	3.44 ± 0.28	2.18 ± 0.34
	norophthalmate	1.69	0.0414	I-P	Ca	0.92 ± 0.32	1.43 ± 0.67	0.29 ± 0.10
	luteolin-*O*-malonylhexoside	1.68	0.0179	I-P; S-P	F	3.13 ± 0.50	3.57 ± 0.28	2.36 ± 0.35
	feruloylquinic acid II	1.54	0.0287	S-I; S-P	H	2.32 ± 0.26	1.51 ± 0.44	1.60 ± 0.16
10 DAF	astilbin	3.75	0.0085	I-P; S-P	F	15.39 ± 6.14	15.41 ± 5.59	0.11 ± 0.12
	caffeic acid-*O*-hexoside II	3.56	0.0012	I-P; S-P	H	8.35 ± 0.93	9.76 ± 3.01	0.04 ± 0.03
	isovitexin	3.28	0.0043	I-P; S-P	F	12.79 ± 3.98	12.25 ± 4.17	0.22 ± 0.08
	chalcone 2′-*O*-glucoside	3.25	0.0035	I-P; S-P	F	10.86 ± 3.56	10.98 ± 3.22	0.12 ± 0.08
	terpenyl-pentosyl-glucoside I	3.10	0.0180	I-P; S-P	M	14.6 ± 5.98	10.33 ± 5.18	0.64 ± 0.25
	phosphoenolpyruvate	2.60	0.0116	S-I; S-P	Ca	1.99 ± 0.60	0.60 ± 0.23	1.03 ± 0.32
	sphinganine	2.58	0.0010	I-P; I-S; S-P	L	1.47 ± 0.15	2.57 ± 0.50	0.66 ± 0.14
	methylsuccinic acid	2.51	0.0059	S-I; S-P	Ca	2.16 ± 0.56	0.81 ± 0.21	1.05 ± 0.18
	kaempferol-3-*O*-rhamnoside	2.31	0.0007	I-P; S-P	F	6.99 ± 1.60	5.01 ± 0.82	0.06 ± 0.04
	dehydroascorbate	1.86	0.0289	S-P	Co.	2.42 ± 0.99	1.21 ± 0.23	0.72 ± 0.38
	linolenoyl ethanolamide	1.85	0.0108	I-P; I-S	L	0.90 ± 0.16	1.53 ± 0.50	0.47 ± 0.09
	3-indoleacetic acid	1.77	0.0035	P-I; I-S; P-S	B	0.47 ± 0.34	1.76 ± 0.43	3.20 ± 0.90
	chrysoeriol-*C*-hexoside-*C*-pentoside	1.73	0.0085	I-P; S-P	F	4.38 ± 1.71	3.17 ± 1.19	0.08 ± 0.03
	chrysoeriol *C*-arabinosyl-*C*-arabinoside	1.69	0.0085	I-P; S-P	F	4.22 ± 1.57	3.05 ± 1.29	0.07 ± 0.02
	phytosphingosine	1.59	0.0013	I-P; S-P	L	1.53 ± 0.21	1.94 ± 0.34	0.65 ± 0.07
14 DAF	lysine	2.68	0.0379	I-P	A	3.02 ± 1.64	4.51 ± 1.41	0.63 ± 0.17
	methionine	2.52	0.0115	I-P; I-S	A	1.93 ± 0.86	3.44 ± 0.55	0.79 ± 0.52
	serine	1.89	0.0101	I-P; S-P	A	2.59 ± 0.61	2.18 ± 0.59	0.52 ± 0.31
	valine	1.84	0.0006	S-I; S-P	A	1.25 ± 0.22	0.42 ± 0.07	0.22 ± 0.06
	histidine	1.56	0.0470	I-P	A	0.93 ± 0.58	1.51 ± 0.44	0.25 ± 0.20
	terpenyl-pentosyl-glucoside II	1.55	0.0092	S-I; S-P	M	2.14 ± 0.07	1.50 ± 0.18	1.33 ± 0.29
	gamma-guanidinobutyric acid	1.53	0.0002	S-P; S-I; I-P	A	1.06 ± 0.13	0.51 ± 0.08	0.16 ± 0.04
	kaempferol-3-*O*-rhamnoside	1.52	0.0337	I-P; S-P	F	0.92 ± 0.24	1.40 ± 0.64	0.14 ± 0.10

^a^ Variable importance in projection (VIP) was obtained from OPLS-DA with a threshold of 1.50; ^b^ False discovery rate (FDR) was calculated from nonparametric Wilcoxon-Mann-Whitney test (one-way ANOVA) with a cutoff of 0.05; ^c^ Post Hoc analyses were performed with Fisher’s least significant difference (LSD); tissue pairs connected with “-” indicate the metabolite level in the former tissue was significantly higher than that in the latter tissue. P: peduncular end, I: intermediate segment, S: stylar end. ^d^ The abbreviations of chemical classes are denoted as follows. A: Amino acid, B: benzene derivative, Ca: Carbohydrate, Co.: Cofactor, F: Flavonoid, H: hydroxycinnamate derivative, L: Lipid, M: Monoterpenol glycoconjugates, N: Nucleotide. ^e^ The relative abundance of metabolite was shown by mean ± SD from four biological replications.

**Table 2:** Example of highly correlated metabolite pairs with biological relations in cucumber fruit.

Metabolite 1	Metabolite 2	Relationship	*r*-Value	*p*-Value
ornithine	citrulline	1	0.992	6.98 × 10^−43^
fumarate	malate	1	0.926	3.98 × 10^−21^
coumaric acid	coumaric acid hexoside	1	0.989	7.96 × 10^−40^
phenylalanine	cinnamic acid	1	0.985	1.72 × 10^−36^
luteolin	luteolin-*O*-malonylhexoside	1	0.978	8.10 × 10^−33^
2-LysoPC(18:2)	2-LysoPC(18:3)	1	0.974	3.17 × 10^−31^
linoleic acid	linolenic acid	1, 3	0.908	5.00 × 10^−19^
chrysoeriol *O*-hexoside I	chrysoeriol *O*-hexoside II	2	0.989	9.32 × 10^−40^
kaempferol	luteolin	2	0.978	6.72 × 10^−33^
tricin 5-*O*-glucoside	tricin 7-*O*-glucoside	2	0.976	3.88 × 10^−32^
kaempferol-3-*O*-glucoside	quercetin-3-*O*-rhamnoside	2	0.949	8.65 × 10^−25^
isorhamnetin-*O*-rutinoside I	isorhamnetin-O-rutinoside II	2	0.935	2.78 × 10^−22^
9,13-DHODE	13(s)-HOTrE	3	0.947	3.02 × 10^−24^
1-LysoPC(18:3)	1-LysoPE(18:3)	3	0.972	1.78 × 10^−30^
1-LysoPC(16:0)	1-LysoPE(18:3)	3	0.961	2.16 × 10^−27^
1-LysoPC(16:0)	1-LysoPE(18:2)	3	0.958	1.19 × 10^−26^
1-LysoPC(16:0)	1-LysoPC(18:2)	3	0.950	8.26 × 10^−25^
1-LysoPC(16:0)	1-LysoPC(18:3)	3	0.950	8.41 × 10^−25^
1-LysoPE(18:2)	1-LysoPE(18:3)	3	0.943	1.11 × 10^−23^
1-LysoPC(18:2)	1-LysoPE(18:2)	3	0.936	1.64 × 10^−22^
1-LysoPC(18:2)	1-LysoPC(18:3)	3	0.926	3.94 × 10^−21^
1-LysoPC(18:1)	1-LysoPC(18:2)	3	0.919	2.97 × 10^−20^
1-LysoPC(18:3)	1-LysoPE(18:2)	3	0.910	3.36 × 10^−19^
1-LysoPC(16:0)	1-LysoPC(18:1)	3	0.903	1.55 × 10^−18^
2-LysoPC(18:2)	2-LysoPC(18:3)	3	0.974	3.17 × 10^−31^
2-LysoPC(18:1)	2-LysoPC(18:2)	3	0.961	2.82 × 10^−27^
2-LysoPC(18:3)	2-LysoPE(18:3)	3	0.923	1.16 × 10^−20^
2-LysoPC(18:1)	2-LysoPC(18:3)	3	0.915	1.03 × 10^−19^

“Relationship 1” indicates that Metabolite 1 and Metabolite 2 are of substrate-product relationship in the same metabolic pathway; “Relationship 2” indicates that Metabolite 1 and Metabolite 2 are isomers with similar MS/MS spectrum; “Relationship 3” indicates Metabolite 1 and Metabolite 2 have similar structure or belong to the same sub-class.

## Supplementary Materials

Supplementary materials can be found at www.mdpi.com/1422-0067/19/1/135/s1.
