# Supplementary material for: Identification of Conserved and Diverse Metabolic Shift of the Stylar, Intermediate and Peduncular Segments of Cucumber Fruit during Development
Source: Int J Mol Sci. 2018 Jan 3;19(1):135. doi: 10.3390/ijms19010135 (PMC5796084; doi:10.3390/ijms19010135)
Supplement: Supplementary file 1 [file ijms-19-00135-s001.zip › Supplementary Figures.docx]

Identification of Conserved and Diverse Metabolic Shift of the Stylar, Intermediate and Peduncular Segments of Cucumber Fruit during Development

Chaoyang Hu^1,2^, Huiyu Zhao^1^, Wen Wang^1^, Mingfei Xu^1^, Jianxin Shi^3^, Xiangbo Nie^4^, Guilin Yang^1,*^

^1^ Lab (Hangzhou) for Risk Assessment of Agricultural Products of Ministry of Agriculture, Institute of Quality and Standard for Agricultural Products, Zhejiang Academy of Agricultural Sciences, Hangzhou 310021, China; zhaohuiyu64@163.com (H.Z.); 411267014@qq.com (W.W); 280971884@qq.com (M.X);

^2^ Key Laboratory of Information Traceability for Agricultural Products of Ministry of Agriculture of China, Institute of Digital Agriculture, Zhejiang Academy of Agricultural Sciences, Hangzhou 310021, China; spiritsun85@163.com

^3^ Joint International Research Laboratory of Metabolic & Developmental Sciences, SJTU-University of Adelaide Joint Centre for Agriculture and Health, School of Life Sciences and Biotechnology, Shanghai Jiao Tong University, Shanghai 200240, China; jianxin.shi@sjtu.edu.cn

^4^ Paojiang Jin Bo Family Farm, Shaoxing 312000, China; 115434002@qq.com

*Correspondence: guilingchina2008@163.com; Tel: +86-571-81999783


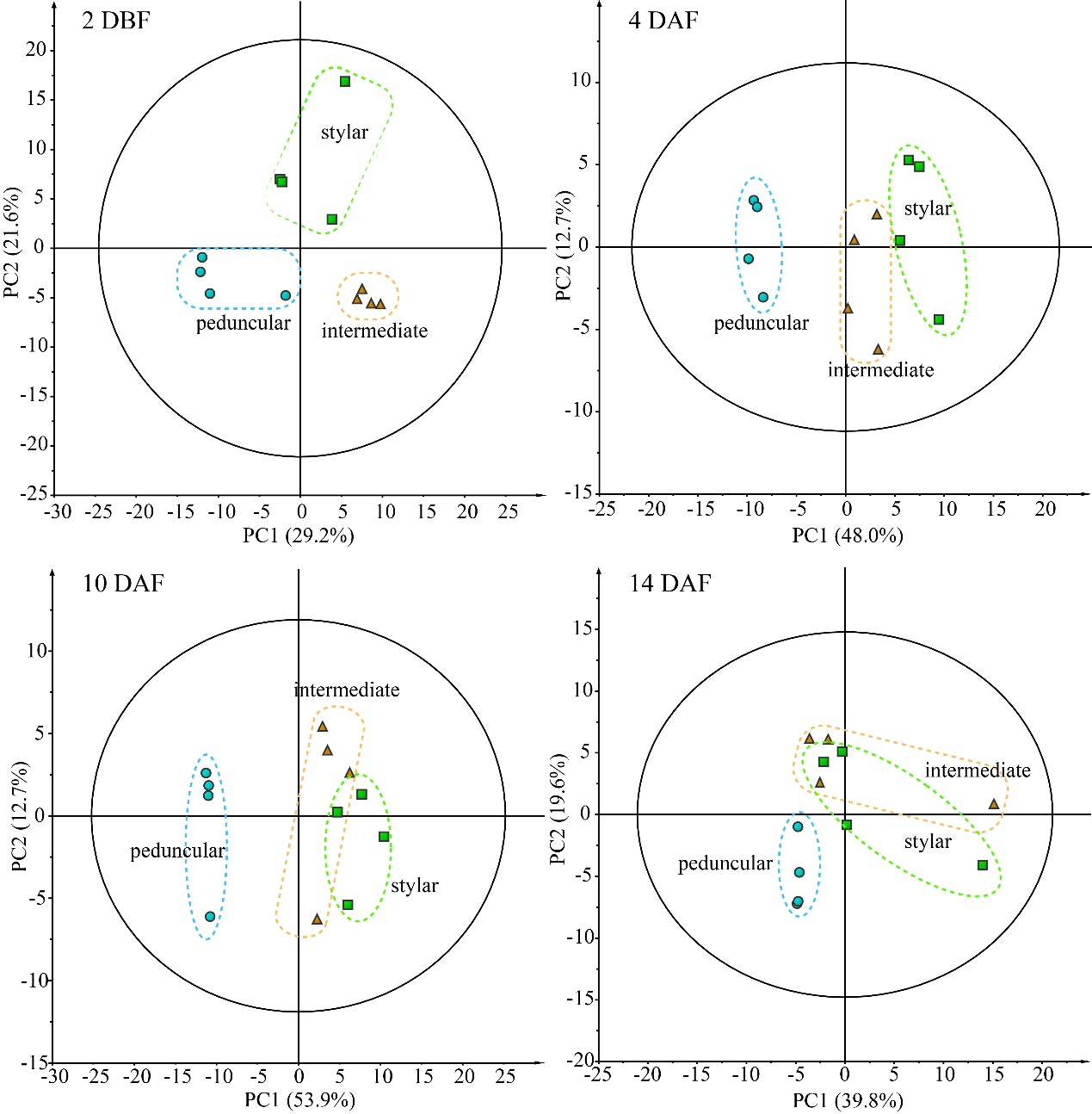


**Figure S1**. Principal component analysis of the metabolomes of stylar end, intermediate segment and peduncular end of cucumber fruit at 2 DBF, 4 DAF, 10 DAF and 14 DAF, respectively.


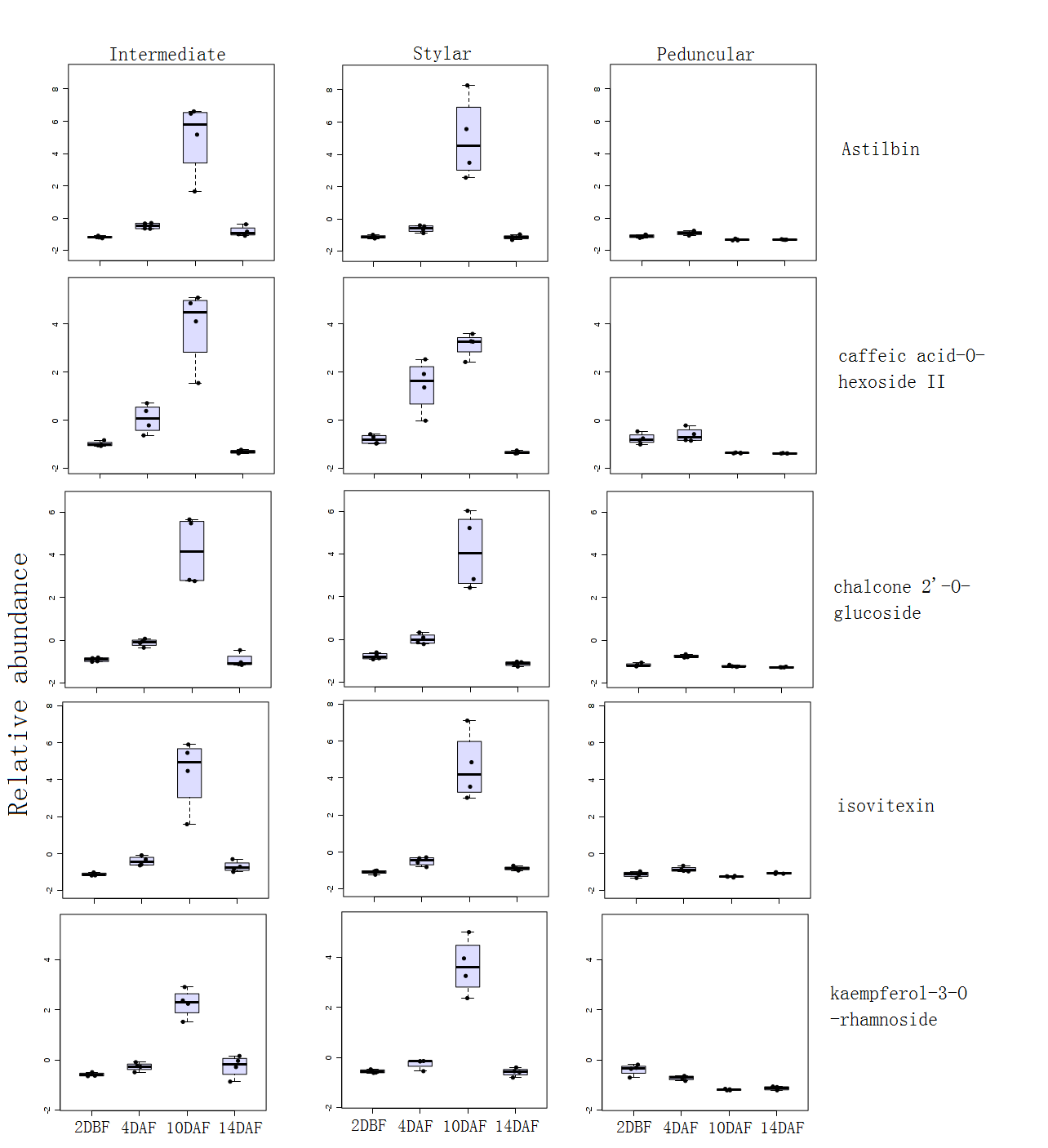


**Figure S2**. Examples of metabolites with diffeential levels among tissues of cucumber fruit.


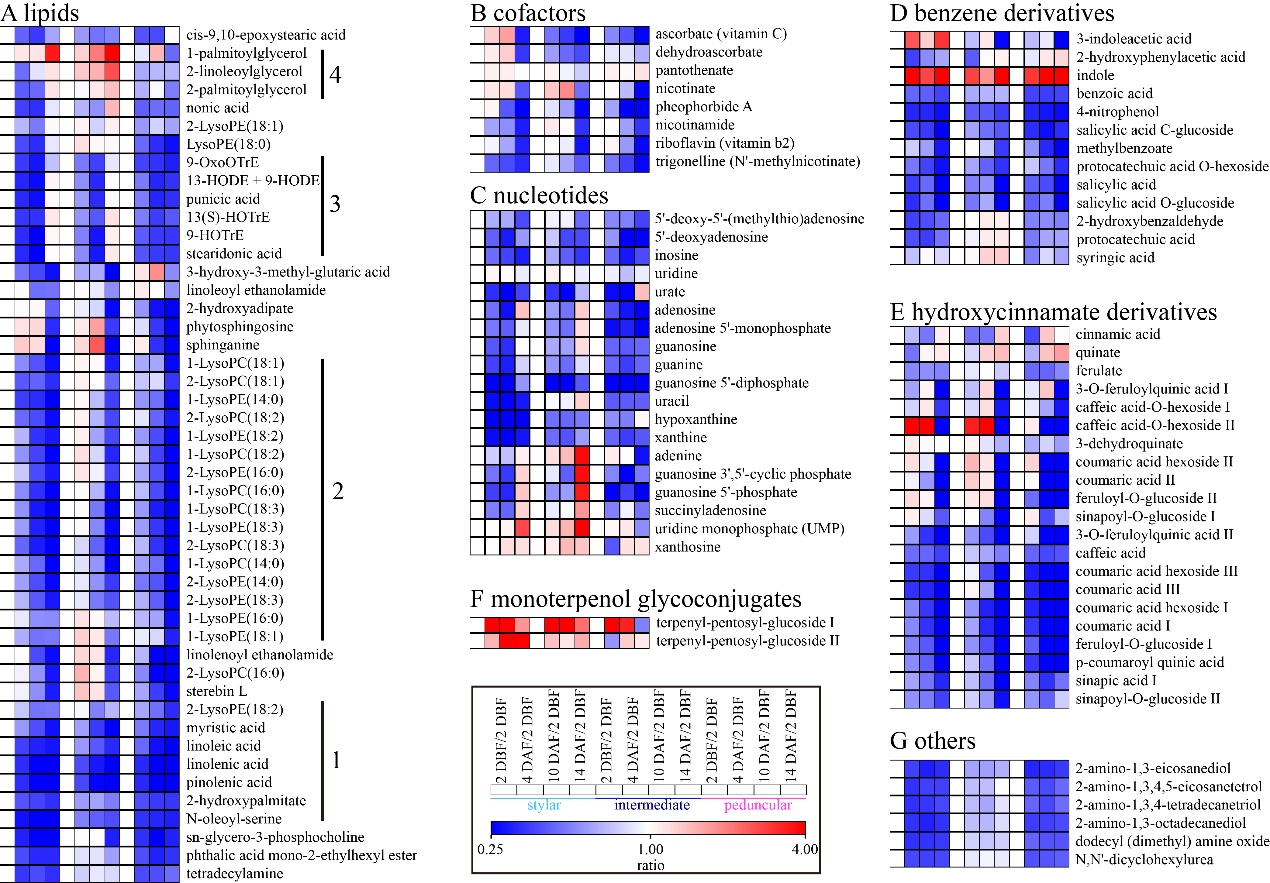


**Figure S3.** Heat map of metabolite changes in stylar end, intermediate segment and peduncular end during cucumber fruit development. Ratios of fold changes were given by shades of red or blue colors according to the scale bar. Ratios were calculated as follows: the mean values from four biological replicates at each time point were divided by those at 2 DBF of the same tissue type to eliminate the tissue-dependent variation.
